# Microchannel Acoustophoresis does not Impact Survival or Function of Microglia, Leukocytes or Tumor Cells

**DOI:** 10.1371/journal.pone.0064233

**Published:** 2013-05-27

**Authors:** Miguel A. Burguillos, Cecilia Magnusson, Maria Nordin, Andreas Lenshof, Per Augustsson, Magnus J. Hansson, Eskil Elmér, Hans Lilja, Patrik Brundin, Thomas Laurell, Tomas Deierborg

**Affiliations:** 1 Neuronal Survival Unit, Department of Experimental Medical Science, Wallenberg Neuroscience Center, Lund University, Lund, Sweden; 2 Department of Measurement Technology and Industrial Electrical Engineering, Lund University, Lund, Sweden; 3 Experimental Neuroinflammation Laboratory, Department of Experimental Medical Science, Wallenberg Neuroscience Center, Lund University, Lund, Sweden; 4 Department of Biomedical Engineering, Dongguk University, Seoul, South Korea; 5 Department of Oncology-Pathology, Cancer Centrum Karolinska, Karolinska Institutet, Stockholm, Sweden; 6 Department of Laboratory Medicine, Lund University, Skåne University Hospital, Malmö, Sweden; 7 Mitochondrial Pathophysiology Unit, Department of Clinical Sciences, Lund University, Lund, Sweden; 8 Departments of Surgery (Urology) and Laboratory Medicine, Memorial Sloan-Kettering Cancer Center, New York, United States of America; 9 Nuffield Department of Surgical Sciences, University of Oxford, Oxford, United Kingdom; 10 Institute of Biomedical Technology, University of Tampere, Tampere, Finland; Duke University Medical Center, United States of America

## Abstract

**Background:**

The use of acoustic forces to manipulate particles or cells at the microfluidic scale (*i.e.* acoustophoresis), enables non-contact, label-free separation based on intrinsic cell properties such as size, density and compressibility. Acoustophoresis holds great promise as a cell separation technique in several research and clinical areas. However, it has been suggested that the force acting upon cells undergoing acoustophoresis may impact cell viability, proliferation or cell function via subtle phenotypic changes. If this were the case, it would suggest that the acoustophoresis method would be a less useful tool for many cell analysis applications as well as for cell therapy.

**Methods:**

We investigate, for the first time, several key aspects of cellular changes following acoustophoretic processing. We used two settings of ultrasonic actuation, one that is used for cell sorting (10 V_pp_ operating voltage) and one that is close to the maximum of what the system can generate (20 V_pp_). We used microglial cells and assessed cell viability and proliferation, as well as the inflammatory response that is indicative of more subtle changes in cellular phenotype. Furthermore, we adapted a similar methodology to monitor the response of human prostate cancer cells to acoustophoretic processing. Lastly, we analyzed the respiratory properties of human leukocytes and thrombocytes to explore if acoustophoretic processing has adverse effects.

**Results:**

BV2 microglia were unaltered after acoustophoretic processing as measured by apoptosis and cell turnover assays as well as inflammatory cytokine response up to 48 h following acoustophoresis. Similarly, we found that acoustophoretic processing neither affected the cell viability of prostate cancer cells nor altered their prostate-specific antigen secretion following androgen receptor activation. Finally, human thrombocytes and leukocytes displayed unaltered mitochondrial respiratory function and integrity after acoustophoretic processing.

**Conclusion:**

We conclude that microchannel acoustophoresis can be used for effective continuous flow-based cell separation without affecting cell viability, proliferation, mitochondrial respiration or inflammatory status.

## Introduction

The use of acoustic forces to handle particles and cells in microfluidic systems (*i.e.* microchannel acoustophoresis) is gaining increased attention [1]. The application in which the acoustophoresis method can be used include particle manipulation [2], [3], depletion [4], washing [5], [6], [7], fractionation [8], rare event sorting [9], [10], concentration [11] and cell cycle synchronization [12]. This novel cell manipulation technique is label-free and enables separation by unique cell properties, *e.g.* compressibility. In view of its high reproducibility, reliability and the fact that this technology can be applied to most cell types, acoustophoresis holds great promise as a cell manipulation technique in several research and clinical settings [13].

While acoustophoresis is emerging as a new technology in several research areas, there are doubts to whether the induced acoustic forces and fluid handling are harmful to the cells. Questions that are relevant to this technology if acoustophoretic applications are to be used with clinical setting. Earlier studies on the impact of acoustic resonant systems on cells have been recently reviewed by Wiklund (2012) [14]. Moreover, Ryll and coauthors studied Chinese hamster ovary cells in a perfused macroscale acoustic cell retention device for 50 days and concluded that no harm was observed to this cell type [15]. In another study, Wang and collaborators studied mouse hybridoma cells, which were acoustically trapped in a high porosity polyester mesh with a low intensity, resonant acoustic field [16], concluded that the acoustic field produced a negligible effect on cell viability in a short-term exposure. Similarly, Hultström and colleagues [17] as well as Evander *et al.* [18], using cos-7 cells from fetal monkey kidney and rat neural stem cells respectively, studied cell viability in an acoustic trap. However, both studies concluded that cell viability was not affected. Moreover, Evander *et al.* successfully grew yeast cells within the trap to demonstrate that cell proliferation was not affected [18].

Although acoustophoretic technology shows great promise, acoustophoretic manipulation of cells in a clinical setting must be studied in detail. Bazou and colleagues studied human liver carcinoma cells (HepG2) in an acoustic trap and determined that cell viability and proliferation were not affected [19]. Using a continuous flow system, Jönsson and coauthors separated erythrocytes from lipid particles and concluded that there had been no increase of hemolysis of erythrocytes after passing through an acoustophoretic device [20]. Recently Dykes *et al.* removed platelets from peripheral blood progenitor cell products by acoustophoresis and cell viability and colony-forming abilities of the progenitor cells was studied. Furthermore, morphological studies as well as platelet activation assays concluded that the cells were not harmed by the acoustophoretic treatment [21]. However, the literature still lacks a thorough examination on the effect of microchannel acoustophoresis using short-term acoustic exposure times with long-term viability and phenotypic characterization. Especially characterizations of important long-term functional biological parameters such as inflammatory response, cell activation response and respiration have not been studied in detail.

If the acoustophoresis technology is used in the clinical setting, the impact on cell survival and the subtle phenotypic changes that may be induced must be investigated in detail. Hence, in this study we examine several key cellular changes following acoustophoresis and use microglial cells, a cell type known to readily react to environmental cues to test cell viability and proliferation. We also monitor more subtle changes in cellular phenotype by analyzing inflammatory status after acoustophoretic processing. Finally, we use similar methodology to examine the effect on human prostate cancer cells and study the different properties of cell respiration using human leukocytes and thrombocytes. Our studies indicate that microchannel acoustophoresis can be tuned for effective cell handling without having any effect of cell viability, proliferation, respiration or inflammatory status.

## Materials and Methods

### Ethics Statement

The study was approved by the regional ethical review board of Lund, Sweden (permit number 113/2008). Blood samples were collected from healthy blood donors at the blood donor central, Skåne University Hospital, Lund. Samples were obtained after written informed consent was acquired.

### Acoustophoresis Device and Setup

**Figure 1:**
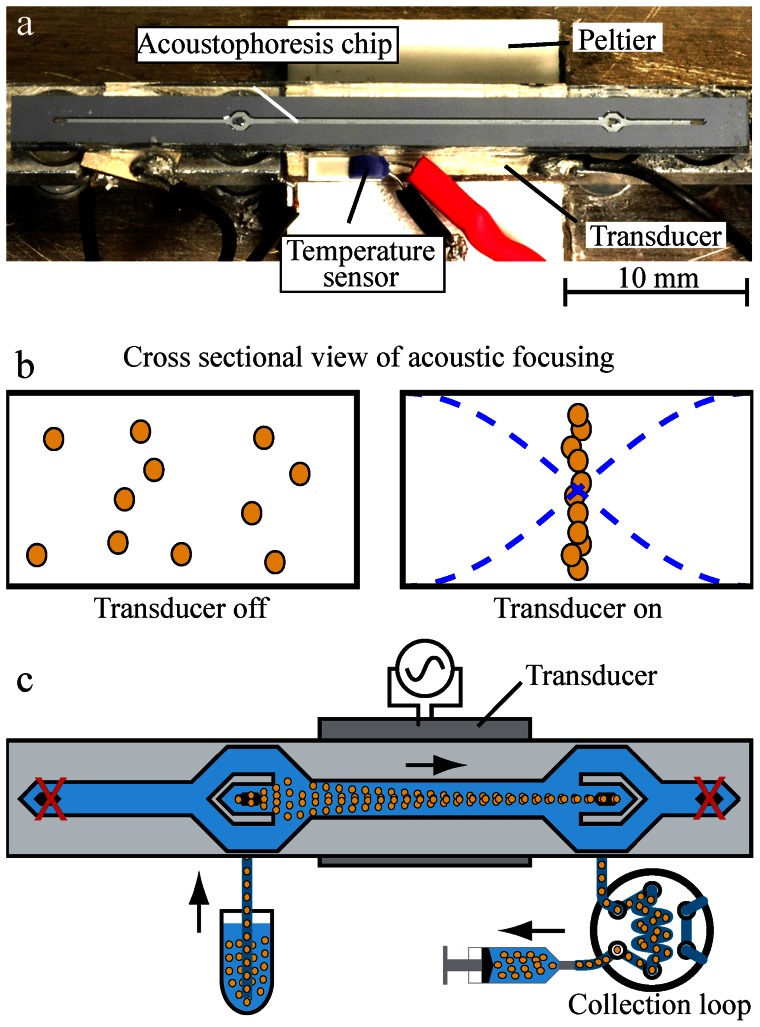
Picture and illustration of the set up. (a) Photo of the acoustophoresis microfluidic system first presented by Augustsson *et al.* [27]. (b) Cross sectional view of cell distribution in the microchannel without ultrasound (left) and with ultrasound forming an ultrasound standing wave (right). (c) Illustration of acouscoustophoresis chip. For the present study, only one of the channel segments was used allowing cells to be exposed to ultrasound.

The acoustophoresis chip design is illustrated in figure 1. The acoustophoresis chip was fabricated in <100>-silicon through double sided photolithography and anisotropic wet etching using KOH. A borofloat glass lid to seal the flow channel was anodically bonded to the chip. The chip structure is comprised of trifurcation inlet followed by a 20 mm long acoustic focusing channel (square cross-section; 375 µm×150 µm) corresponding to a retention time of about 1 second in the acoustic field for a flow rate of 100 µL min^−1^ and a trifurcation outlet region. For the viability experiments only one inlet and outlet was used. The center outlet flow rate was controlled by a syringe pump (WPI sp210iwz, World Precision Instruments Inc., Sarasota, FL, USA) and set to 100 µL min^−1^ and the cell suspension was drawn into the center inlet from an Eppendorf tube at the same speed. To avoid sedimentation in the tube during the course of the experiments the cells were mixed gently by pipetting. Samples were collected either by a 6-port 2-way sample loop (V-451, Upchurch Scientific, Oak Harbor, WA, USA) with a volume of 100 µL connected in series with the outlet or directly in the syringe (for the human blood cell respiration studies). To actuate the chip a piezoceramic transducer (PZ26, resonant at 2 MHz, Ferroperm, Kvistgaard, Denmark) was used. The piezoceramic was driven by a wave form generator (Agilent, 33250A) set at a frequency of 1.94 MHz, amplified by an in-house-built amplifier. The voltage over the transducer was measured using an oscilloscope (TDS 1002, Tektronix UK Ltd., Bracknell, UK). The temperature of the chip was controlled using a Peltier element regulated by a Peltier regulator (TC2812-RS232, CoolTronic GmbH, Beinwill am See, Switzerland) and set to 37°C to limit effects emanating from elevated temperature exposure caused by power dissipation in the piezo ceramic at elevated operating voltage that could affect the viability of the cells. The cells were processed through the chip and exposed to ultrasound at either 10 or 20 V_pp_ operating voltage or run through the chip without ultrasound exposure to reveal any influence of the microfluidic system. Unprocessed cells were used as a control. The acoustic energies the cells were exposed to were higher than would normally be needed to focus cells in this chip for the particular settings stated above. In this set up, thrombocytes were less prone to focus in the middle of the microchannel due to their small sizes.

### Estimation of the Acoustic Energy Density

To estimate the acoustic energy density (*E*_ac_) inside the acoustophoretic microchannel polystyrene microparticles (∼0.02%_wt_, ∅ 7 µm) (Sigma-Aldrich. Co., St. Louis, MO, USA) suspended in Triton X-100 (0.01%) in PBS were processed in the device. To estimate *E_ac_* the microparticles were drawn into the chip from the side inlets at flow rate of 50 µL min^−1^ and a clean buffer was pumped into the center inlet at a flow rate of 450 µL min^−1^. The beads were then focused in the center of the channel and collected from the center outlet at a flow rate of 250 µL min^−1^ using two 6-port 2-way sample loops (V-451, Upchurch Scientific, Oak Harbor, WA, USA), each with a volume of 100 µL. Excess fluid was discarded from the side outlets also at a flow rate of 250 µL min^−1^.

#### Material

The fluorescent marker for DNA 7-Aminoactinomycin D (7-AAD), the synthetic androgen agonist methyltrienolone (R1881), 2,3-Bis(2-methoxy-4-nitro-5-sulfophenyl)-2*H*-tetrazolium-5-carboxanilide(XTT), Giemsa stain, 2′-(4-hydroxyphenyl)-5-(4-methyl-1-piperazinyl)-2,5′-bi-1*H*-benzimidazole trihydrochloride hydrate, bisBenzimide (Hoechst), and Lipopolysacharide (LPS from *Escherichia coli*, serotype 026:B6) were purchased from Sigma Aldrich (Stockholm, Sweden). Tetramethylrhodamine, ethyl ester, perchlorate (TMRE) was purchased from Invitrogen (Stockholm, Sweden). Cytokines were detected using a Multiplex kit from Meso Scale Discovery (Gaithersburg, USA).

#### Cell culture

The murine microglial BV2 cells were a kind gift of Dr. Sandra Ceccatelli and were established and first characterized 1992 by Bocchini *et al.* [22]. All Cell lines were culture in DMEM (GIBCO) and supplemented with 10% fetal bovine serum (FBS) (Sigma-Aldrich), 55 IU ml^−1^ penicillin and 55 µg ml^−1^ streptomycin (Sigma-Aldrich). For the experiments performed with the BV2 cells and LPS, the media was supplemented with 5% FBS. All cells were cultured at 37°C in a humidified atmosphere containing 5% CO_2_. The human prostate cancer cell lines DU145, LNCaP, PC3 and VCaP were all obtained from the American Type Culture Collection (ATCC) and grown according to ATCC recommendations.

#### Cell viability in BV2 cells

The BV2 microglial cells were suspended (4 million cells mL^−1^) in culture medium supplemented with 2% serum to facilitate phenotypic/inflammatory changes of the cells. After the acoustophoretic processing possible induction of acute cell death was examined by dye exclusion using trypan blue.

#### XTT assay

5000 BV2 cells per well were seeded in 96-well plates after microchannel acoustophoretic processing and cultured for 24 or 48 h. The XTT assay was performed following the manufacture’s recommendation and the data are presented as percentage in respect to the cells exposed to microfluidic handling without ultrasound.

#### TMRE staining

20,000 BV2 cells were seeded in 12-well plates to measure their Mitochondrial potential (ΔΨ) after acoustophoretic processing. A final concentration of 25 nM of TMRE staining was added to the medium and the fluorescence was measured using flow cytometry (FACS CantoII, FACSDiva software, BD Biosciences, San Jose, USA).

#### Cell migration

BV2 cells were seeded in 6-well plates after acoustophoretic processing. Twenty-four hours after seeding, a yellow 100-µl-pipette tip was used to draw a line through the confluent culture dish. After additional 24 h of culture, the width of the line, *i.e.* how much cells had migrated, was analyzed.

#### Hoechst staining

BV2 cells were seeded in 12-well plates and fixed in 4% PFA during 20 minutes at 37°C. The plates were then washed twice with PBS and Hoechst was added at a concentration of 1 µg mL^−1^ in PBS for 10 min at room temperature. After two more washes with PBS, mounting media was added to the wells and the number of cells that presented condensed nuclei was quantified using an inverted microscope. For each well at least 300 cells were counted.

#### Clonogenic assay

1000 BV2 cells were seeded in duplicates in petri dishes and 10 mL of medium was added to each dish. After three days, 5 mL of medium was exchanged with fresh media. After seven days the media was carefully removed and the cells were fixed with 70% ethanol during 10 min at room temperature. After the fixation the cells were stained with 10% (v/v) GIEMSA staining for 15 min at room temperature. The plates were then washed with MilliQ water and dried after which they were scanned and analyzed using ImageJ 1.43r software.

#### Immunoblot

After acoustophoretic processing, BV2 cells were seeded in 6-well plates and after one day exposed to LPS for 24 h. The cells were then washed twice in PBS and lysed on ice using loading buffer. Then, the cells were sonicated 3 times during 5 seconds each time while kept on ice. All samples were boiled for 4 min, resolved on 10% SDS polyacrylamide gels at 100 V and transblotted onto nitro-cellulose membranes for 2 h at 30 V. The membranes were blocked for at least 1 h in a buffer (5% non-fat milk powder and 0.1% NaN3 in PBS, pH 7.4) and probed with primary antibodies (diluted in 5% bovine serum albumin and 0.1% NaN3 in PBS, pH 7.4) against iNOS (Santa Cruz, sc-650; 1∶1000) and β-actin (Sigma, A5316 1:4000) overnight. The membranes were then washed in PBS containing 0.1% Tween-20 and incubated with the secondary antibody (diluted 1∶5000 in 2.5% milk in PBS) for 1 h and washed 3 times in PBS. The protein bands were visualized by ECL (Amersham Biosciences) according to the manufacturer’s instructions.

#### Cytokine analysis

After acoustophoretic processing, BV2 cells were seeded in 96-well plates. After 24 h, cells were exposed to LPS for 24 h and the supernatants were collected. The cytokine production was measure using the Meso Scale Discovery electrochemiluminescence kit (Th1/Th2 cytokines, Ultra-Sensitive kit, K15013C-1) using the manufacturer’s instructions.

#### Cell viability in prostate cancer cell lines

DU145, PC3, LNCaP and VCaP cells were detached by trypsin/EDTA and washed twice in PBS and re-suspended in complete cell growth medium. Immediately after acoustophoretic processing (0 h) trypan blue exclusion was used to estimate the percentage of viable cells. The cells were counted in a Bürker chamber and afterward seeded in 6-well plates, (200,000 cells/well) and grown for 24 or 48 h. Cells were harvested with trypsin/EDTA, washed with PBS and subsequently stained with 7-AAD for 20 minutes. Cell viability was estimated for 10,000 cells by flow cytometry (FACS CantoII, FACSDiva software, BD Biosciences), 7-AAD-negative cells with high forward scatters were selected as viable.

#### PSA secretion

Cell lines (LNCaP and VCaP) expressing the androgen receptor (AR) were harvested and subjected to the acoustophoretic processing. Unprocessed cells were used as a control group. Cells were seeded in 96-well plates (10,000 cells/well), and grown in complete growth medium for 24 h in the presence of either R1881 (1 nM), or vehicle (PBS/DMSO). 80 µL growth medium was removed from each well for PSA analysis. The samples were stored at −20°C until analysis. PSA concentrations were measured using the DELFIA Prostatus Free/Total PSA assay from PerkinElmer, Turku, Finland [23].

#### Human blood cell respiration

Thrombocytes and leukocytes from three healthy blood donors were subjected to acoustophoretic processing and mitochondrial function was analyzed by high-resolution respirometry as described in Sjövall *et al.* [24].

#### Statistics

We used analysis of variance (ANOVA) for statistical comparisons between experimental groups. Experiments were performed in triplicates or more. Data are expressed as mean ± SD and P<0.05 was considered as significantly different.

## Results

### Acoustic Energy Estimations

To estimate the acoustic energy (*E_ac_*) at the different driving voltages, we used 7 µm diameter microparticles. Microparticles were used instead of cells do to their homogenous size distribution, which allows for a better measurement. The driving voltage squared (*U*_pp_)^2^ has a linear relation to the *E*_ac_ in the following expression;
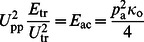
where *U*_tr_ is the transition voltage applied when the transition from particles exiting through the side outlets to exiting from the center outlet takes place, *E*_tr_ is the corresponding acoustic transition energy, *p*_a_ is the acoustic pressure amplitude and κ_o_ is the compressibility of the suspending liquid. For this particular chip, using the 7 µm polystyrene microparticles and a total flow rate of 500 µL min^−1^, U_tr_^2^ was measured to be 147 V_pp_^2^ and *E*_tr_ was then estimated from simulated particles trajectories to be 69 J/m^3^. Through this expression *E_ac_* for the voltages of 10 V_pp_ and 20 V_pp_ was calculated to be 47 J/m^3^ and 188 J/m^3^, which correspond to acoustic pressure amplitudes of 0.639 MPa and 1.28 MPa, respectively. These energy levels are comparable to other acoustophoresis microchip processing devices, which commonly operate at acoustic energy density levels of 10–100 J/m^3^ [25], [26]. See Augustsson *et al*. 2012 for a more detailed description of the acoustic energy density estimation [27].

### Acoustic Cell Processing

Microchannel acoustophoretically processed cells may be damaged due to hydrodynamic shear, ultrasonic exposure or high temperatures. To avoid cell damage due to high temperatures, we evaluated the effect of acoustophoresis on cell viability in a controlled temperature environment (37°C). Hydrodynamic shear damage was also excluded by using cells processed through the chip without ultrasound exposure as controls.

In the acoustophoresis microchannel, cells were focused in the center when operated at a half wavelength resonance at 2 MHz. At amplitude of 10 V_pp_, all cells were focused in the center of the microchannel. Amplitude of 20 V_pp_ was also employed to generate a stronger acoustic field than needed to focus cells, and thereby enable the study of possible harmful effects on the cells when exposed to elevated acoustic radiation forces. The setup allowed maximum 25 V_pp_, however using this high voltage generated temperature fluctuations above the temperature criteria of 37°C. We tested the effect 25 V_pp_ on immediate cell death (trypan blue exclusion) using the BV2 microglial cells without detecting any adverse effect (data not shown). As a control, cells were processed through the chip without an active ultrasound (function generator set to 0 V_pp_). In order to study the effect of acoustophoresis on cell survival and elucidate possible adverse effects when using acoustophoresis for cell sorting, a standard acoustophoresis chip was used (fig. 1, described in Material and Methods). Cells were passed through the chip at a flow rate of 100 µL min^−1^ corresponding to a retention time of about 1 second in the acoustic field, a retention time relevant for most acoustophoretic processing.

### Acoustic Cell Processing in a Microchannel does not Alter Cell Death and Viability of Microglial Cells

#### Viability and cell death analysis

We set out to investigate possible deleterious effects of acoustophoresis by using the microglia cell line BV2, an immortalized mouse microglial cell used widely and successfully in medical research [28]. Using trypan blue exclusion, no adverse effects on the acute cell death induced by the acoustophoresis handling could be detected (Ctrl, cells not entering the acoustophoresis chip 9.7±5.7% cell death; 0 V_pp_, 11.4±8.8% cell death; 10 V_pp_, 8.9±7.0% cell death; 20 V_pp_, 11.6±6.9% cell death).

**Figure 2:**
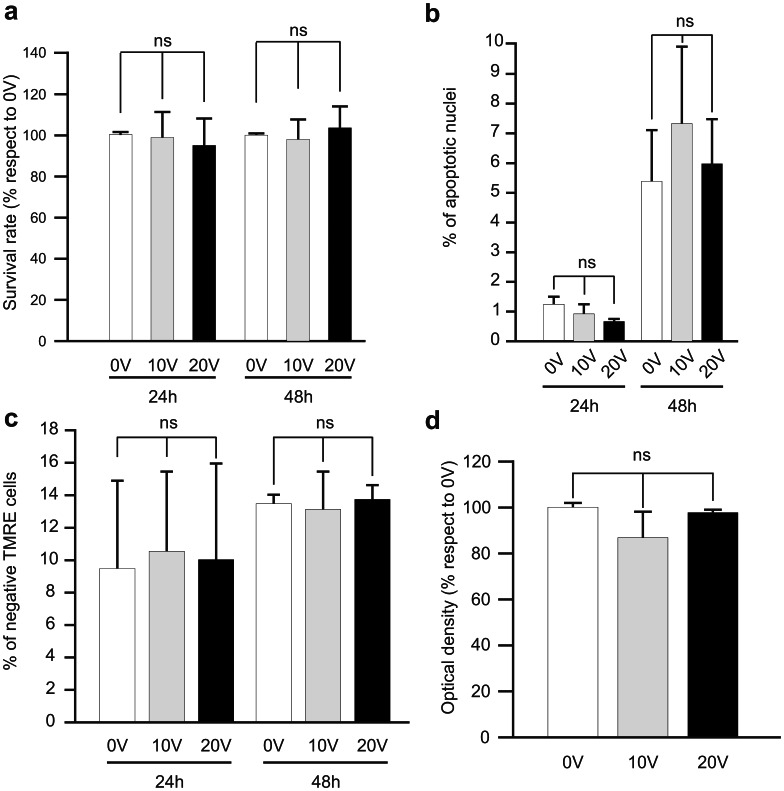
Unaltered viability of BV2 microglial cell line following acoustophoretic processing (10V_pp_ and 20V_pp_). BV2 cells were passed through the acoustophoresis chip with the function generator set at 0, 10 and 20 V_pp_. After going through the chip, the BV2 cells were seeded again for 24 and 48 h. Cell viability was measured by XTT (a), apoptotic nuclei appearance (b) and decrease of mitochondrial potential -Ψm- (c), showing no difference between experimental groups. Similar, no difference was detected by clonogenic assay (d) used to study survival and proliferation at 7 days following acoustophoretic processing. The graphs show the results from at least three separate experiments and the data are shown as means ± SD. Significance value *P*<0.05, ns denotes non-significant.

Next, we studied possible long-term cellular alterations beyond the acute effect of acoustophoresis. After going through the acoustophoresis chip, BV2 cells were seeded again for 24 h or 48 h to investigate possible long-term effects. First, we used XTT assay (mitochondrial dehydrogenase activity) to study a possible alteration in the viability/proliferation of acoustophoresis. However, no differences in viability of the cells exposed to the different conditions could be detected in the XTT assay at either 10 or 20 V_pp_ of acoustic focusing at any time point, 24 or 48 h, after seeding (fig. 2a). To further elucidate the effect of acoustophoresis, BV2 cells were PFA-fixed then Hoechst stained at 24 and 48 h and the number of cells undergoing cell death, containing apoptotic bodies, were quantified. Despite a progressive cell death from 24 to 48 h, we did not find any changes in number of cells undergoing apoptosis due to the acoustophoretic processing (fig. 2b). Furthermore, we did not detect any changes in the mitochondrial potential (indirectly measurement of cells at risk of undergoing apoptosis) by TMRE due to acoustophoresis (fig. 2c). Similarly, the cell migration speed as described previously [29], was unaltered after acoustophoretic processing at 24 to 48 h after culturing (data not shown). The cellular changes over a longer time period (7 days) was also assessed to study a possible alteration in cell turnover using a clonogenic assay however, no adverse effect were detected (fig. 2d) similar to our earlier time points (24 or 48 h).

#### Inflammatory response

**Figure 3:**
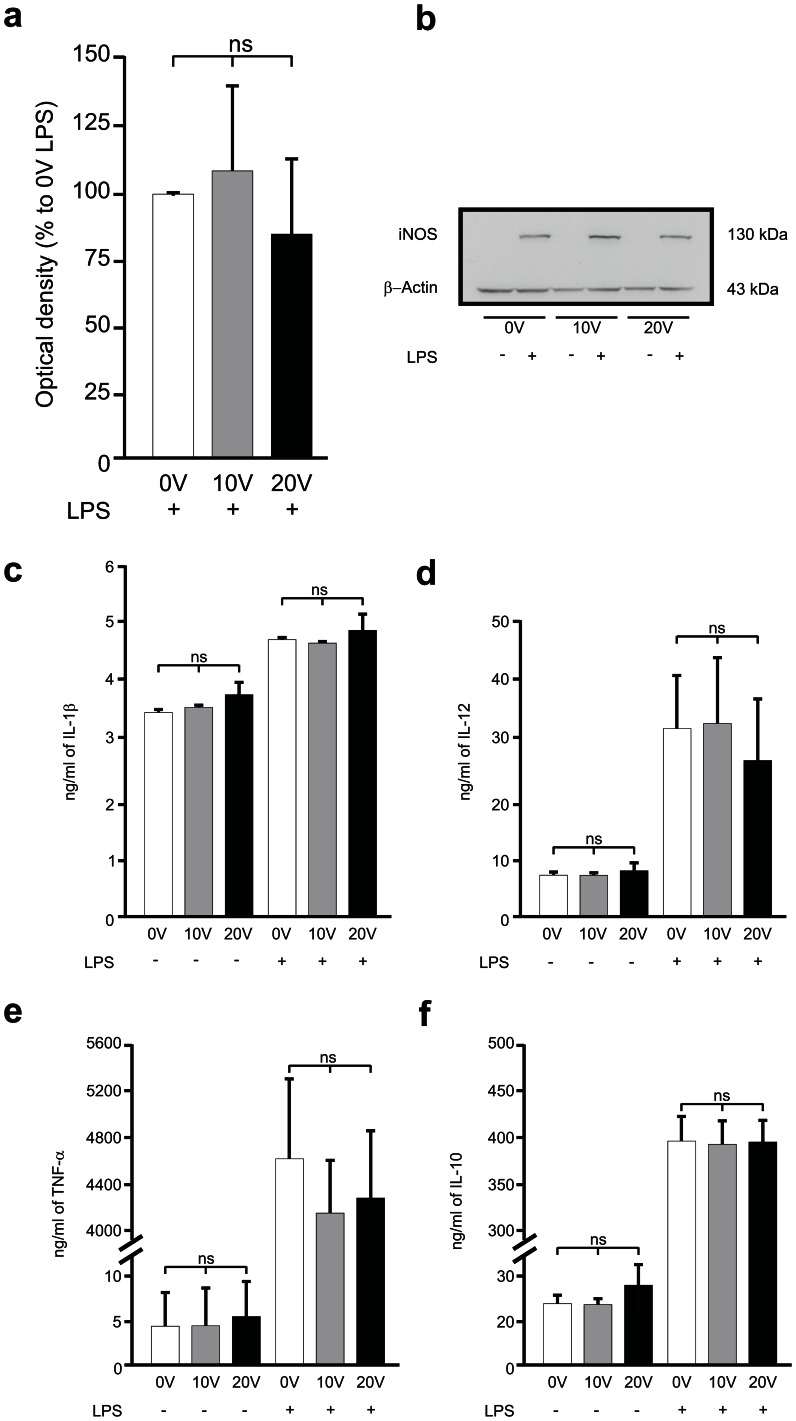
Inflammatory response of BV2 cells upon LPS challenge following acoustophoresis is not changed. After acoustophoretic processing, BV2 cells were seeded and stimulated the next day with LPS for 24 h. We observed no alteration due to acoustophoretic processing in the expression of iNOS (inducible nitric oxide synthase)(a,b), the release of proinflammatory cytokines IL-1β (χ), IL-12 (d), TNF-α (e) or the anti-inflammatory cytokine IL-10 (f). The graphs show the results from at least three separate experiments and the data are shown as means ± SD. Significance value *P*<0.05, ns denotes non-significant.

Although we could not detect any acoustophoresis-induced alterations on cell death or cell viability, minor cellular alterations and phenotypic changes cannot be ruled out. In this aspect, inflammatory microglial cells are suitable cells to detect subtle differences in their inflammatory response due to the exposure of acoustic forces. We therefore explored the possibility that acoustophoretic processing could alter the inflammatory response triggered by a proinflammatory response. Lipopolyssacharide (LPS) is a lipoglycan found in the outer membrane of Gram-negative bacteria and a very potent Toll-like receptor 4 (TLR4) ligand and inducer of inflammation. LPS-challenged BV2 cells were analyzed for the production of inducible nitric oxide synthase, iNOS, (fig. 3a,b) that is highly induced under inflammatory stimulus (such as LPS). The medium was also collected and used to measure the microglial release of proinflammatory cytokines (IL-1β, IL-12 and TNF-α; fig. 3c,d,e) and anti-inflammatory molecules (IL-10; fig. 3f). We found that, both the protein expression of proinflammatory iNOS and the release of pro- and anti-inflammatory cytokines upon LPS-challenge were unaltered after exposure to acoustophoretic processing (fig. 3).

### Influence of Acoustophoresis on Viability or Secretory Function of Prostate Cancer Cells

**Figure 4:**
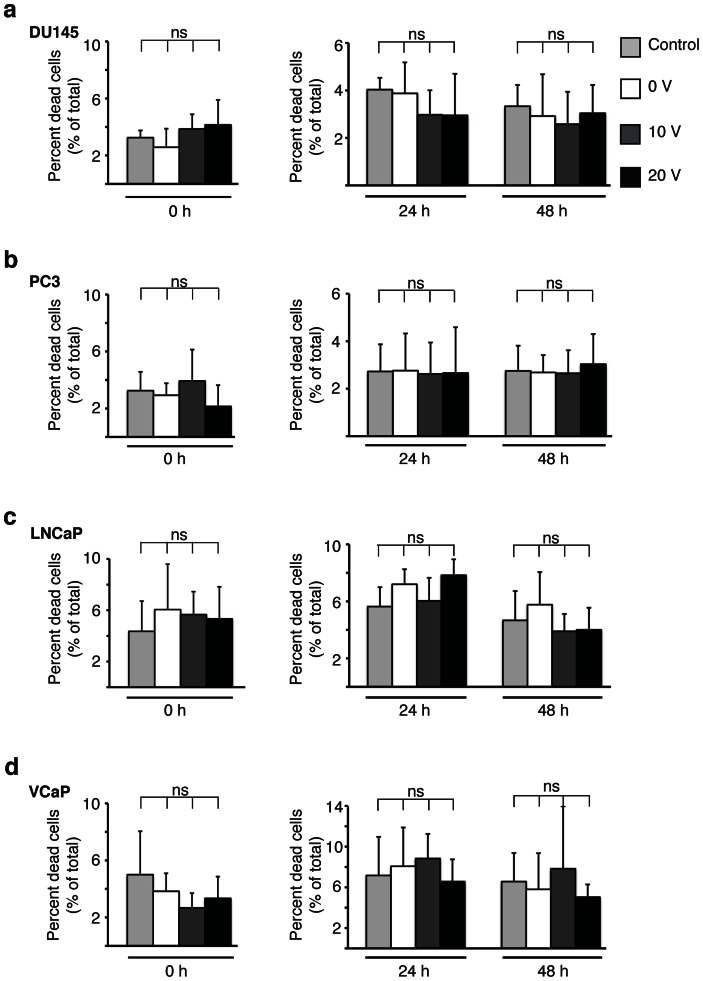
Prostate cancer cell viability is not affected by acoustophoresis. Cell viability was determined after acoustophoresis (0, 10 and 20 V_pp_), for four prostate cancer cell lines: DU 145 (a), PC3 (b), LNCaP (c) and VCaP (d). Left panels show the percentage of cell death directly after acoustophoresis measured by trypan blue exclusion. Right panels show the cell death quantified at 24 and 48 h after acoustophoresis by flow cytometry. Cells negative for the DNA binding fluorochrome 7-Aminoactinmycin (7-AAD) were defined as viable. At least 10,000 cells were counted and the percentage of dead cells was determined. Cells not subjected to acoustophoresis were used as control cells. The graphs show the results from at least three separate experiments and the data are shown as means ± SD. Significance value *P*<0.05, ns denotes non-significant.

Acoustophoresis-based microfluidic devices are currently being explored as a mean to enrich and discriminate viable from non-viable tumor cells in peripheral blood [27]. Therefore, we initiated a detailed characterization of the viability and secretory function of tumor cells subsequent to subjecting them to acoustophoretic processing. We first characterized the prostate cancer cell lines DU145, PC3, LNCaP in reference to cell viability/proliferation using the same XTT assay that was used for the viability test of the BV2 microglial cells. We did not find any changes in cell viability at either 24 or 48 h after acoustophoretic processing (data not shown). Subsequently, we determined the extent of cell death using trypan blue exclusion directly following acoustophoretic processing without observing any change in cell death for the different prostate cancer cell lines, DU145, PC3, LNCaP or VCaP. Finally, we examined cell death at 24 and 48 h using florescence activated cell sorting (FACS) and gated cells for 7-AAD-uptake. Again, similar to our results above, no adverse effect following acoustophoretic processing when compared to unprocessed cells was identified (fig. 4a–d).

**Figure 5:**
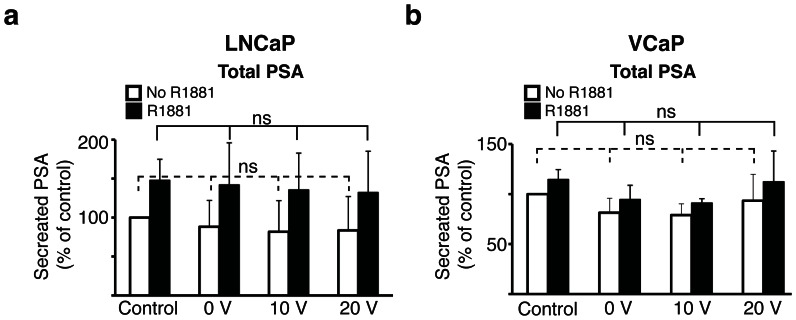
Acoustic cell separation in a microchannel does not alter PSA secretion by prostate cancer cells. The androgen receptor (AR) expressing cell lines LNCaP and VCaP were used to evaluate the impact of acoustophoresis on PSA secretion. After acoustophoresis run at 0, 10 and 20 V_pp_, the secretion of PSA was measured in the absence or presence of the AR ligand R1881 (1 nM for 24 h) in the LNCaP cell line (a) and in the VCaP cell line (b). Cells not processed through the chip were used as control cells. The graphs show the results from three separate experiments and the data are shown as means ± SD. Significance value *P*<0.05, ns denotes non-significant.

We went on to determine whether acoustophoresis alters the functional properties of viable cells processed through the microchip. PSA secretion is a sensitive indicator for androgen receptor (AR) signaling activity in AR-dependent tumor cells. We used the prostate cancer cell lines LNCaP and VCaP and studied both endogenous PSA secretion as wells as secretion following administration of the synthetic androgen R1881. We did not find any significant differences in PSA secretion in tumor cells subjected to acoustophoretic processing compared to unprocessed cells, neither after administration of R1881 nor for unstimulated cells. There were no measurable differences in reference to cells processed through the microchip in absence of ultrasound or when they were exposed to ultrasound at increasing voltage (fig. 5a–b).

### Mitochondrial Respiratory Function of Human Blood Cells is not Altered Following Acoustophoresis

Acoustophoretic processing of human blood is an emerging research field of acoustic cell manipulation in microchannels. To further elucidate possible cellular alteration due to acoustophoresis, we exposed thrombocytes and leukocytes isolated from human blood to the same acoustic treatment used for the BV2 microglial cells and prostate cancer cells described above. High-resolution respirometry was performed to evaluate mitochondrial respiratory function and integrity. Furthermore, endogenous oxygen consumption was evaluated in intact cells (data not shown), and respiration during maximal oxidative phosphorylation (Oxphos) and maximal flux through the electron transport system was assessed using a multiple substrate/inhibitor titration protocol in digitonin-permeabilized cells.

**Figure 6:**
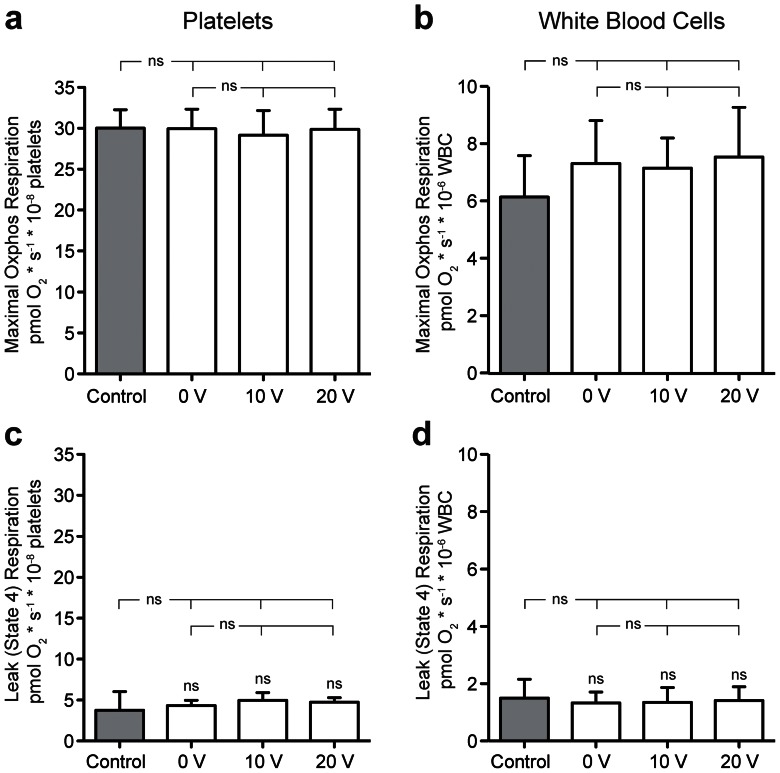
Mitochondrial respiratory function in human leukocytes and thrombocytes are not altered following acoustophoresis. Leukocytes and thrombocytes were passed through the acoustophoresis chip run at 0, 10 and 20 V_pp_. Maximal respiration during using both complex I- and complex II-linked substrates for thrombocytes (a) and leukotcytes (b) was unaltered after acoustophoresis, supporting that acoustophoresis does not affect metabolic pathways important for respiration. The remaining respiratory activity following inhibition of ATP synthase with oligomycin, so called Leak or state 4 respiration, was also unaffected by acoustophoresis, in thrombocytes (c) and leukocytes (d). This data confirm no effect of acoustophoresis on inner mitochondrial membrane integrity or changed utilization of proton motive force for other purposes than ADP phosphorylation. Unprocessed cells were used as control cells. The graphs show the results from three separate experiments and the data are shown as means ± SD. Significance value *P*<0.05, ns denotes non-significant.

We studied the maximal respiration during Oxphos using both complex I- and complex II-linked substrates for thrombocytes (fig. 6a) and leukocytes (fig. 6b) and found no effect following acoustophoretic processing. We further investigated the remaining respiratory activity following inhibition of ATP synthase with oligomycin, so called LEAK or state 4 respiration, and found no differences in respiratory activity following passage through the acoustophoresis microchip. We also checked for differences in other respiratory states, without detecting any alterations due to acoustophoretic processing (data not shown). Any process interfering with metabolic pathways leading to respiratory inhibition would be readily detected at maximal Oxphos respiration. Similarly, any process interfering with inner membrane integrity or increased utilization of proton motive force for other purposes than ADP phosphorylation would be readily detected at LEAK/state 4 respiration. Thus, there is no indication of any negative mitochondrial effect or an increase in energy demand in the cells by acoustophoretic cell sorting.

## Discussion

The acoustophoretic technology allows non-contact cell handling based on intrinsic cell properties that includes size, density and compressibility which enables the development of novel label-free cell sorting strategies [27]. However, extensive analysis on the effects of sorting methodology are necessary not only for a technique to be established in clinical settings but also to assure that research results do not get tainted due to a harsh sorting technology. For a cell handling or sorting technologies to be valid for clinical or research purposes, they need be non-perturbing for the cells processed. Hence, in this study we have performed a comprehensive study with different cells using various techniques aimed at determining any cellular alterations that acoustophoretic processing could induce. Our conclusions in this study are relevant for acoustophoretic sorting of cells in microfluidic systems at flow rate in the range up to 100 µl/min (channel cross-sections 375*150 µm) and with retention times in the acoustic standing wave for about 1 second, at pressures up to 1.28 MPa, at an actuation frequency of 2 MHz.

The effect of acoustophoretic processing on microglial cells was first elucidated. These cells are the inflammatory surveillance cells of the brain similar to macrophages in other tissues [30]. Microglia respond easily to subtle changes in the microenvironment, including exposure to radiation [31], nanoparticles [32] and minor temperature changes [33]. The murine microglial cell line BV2 is an inflammatory cell line widely used in medical research [28]. We first used this cell line and found no changes in survival/apoptosis proliferation or migration at 24 h, 48 h or 7 days after acoustophoretic processing using both manual cytological analysis and flow cytometry (TMRE). After demonstrating that acoustophoresis did not alter aspects of viability, we further determined if acoustophoresis could induce any changes in the inflammatory state of the cells. Microglia exposed to acoustophoretic processing were also stimulated with LPS activating the pattern recognition receptor TLR4. This receptor is known to activate the MyD88 or TRAM/TRIF pathways. Stimulation of these pathways will trigger intracellular pathways activating inflammatory transcription factors (IRF/NFkB/CREB/AP-1) that will lead to iNOS activation and cytokine release. Our data show no alteration in iNOS and cytokine response following acoustophoretic processing suggesting that these inflammatory pathways are not affected by acoustophoresis. In view of the susceptible activation of microglial cells by various stimulus, our data suggest that acoustophoresis is a mild cell-handling technology.

Prostate cancer is the most commonly diagnosed cancer in men and has the second highest death incidence [34]. However, the molecular background of prostate tumor progression is currently not completely understood. Most prostate cancer cells require androgen stimulation for growth and proliferation and androgen deprivation therapy is the first line of treatment for most prostate cancer patients with disseminating disease. Although effective in reducing tumor growth and size, the treatment selects for androgen independent cancer cells, resulting in tumors resistant to androgen therapy and progression into castration-resistant prostate cancer [35]. Molecular interrogation of isolated circulating tumor cells would provide valuable information on tumor progression and metastasis. It is crucial for future cell evaluation, that the separation technology’s intrinsic properties do not alter any physical appearances or functional properties of the processed cells. Here, we demonstrate that acoustophoresis affects neither the responsiveness nor the endogenous PSA secretion or PSA secretion induced by R1881 (a synthetic androgen), in prostate cancer cells. Acoustophoresis may therefore be a suitable technique for live CTC isolation from blood.

Mitochondria generate most of the cell’s supply of ATP through oxidative phosphorylation. ATP is the main energy source for the majority of cellular functions, including signal transduction pathways, maintenance of cell structure, ion homeostasis and locomotion. Any external influence on these cellular processes leading to increased ATP demand will directly influence mitochondrial ATP production rate. Acoustophoresis could thus potentially influence mitochondrial activity indirectly by increasing ATP production demand. Mitochondrial dysfunction is implicated in numerous disorders [36] and is a common cause for drug-induced toxicity [37]. An altered capacity of oxidative phosphorylation may affect energy supply to the cell, redox state and handling of reactive oxygen species, as well as apoptotic cell death pathways [38]. Measuring cell respiration is an integrated approach to assess mitochondrial function in cells [39]. Oxygen consumption is directly proportional to the flux through the electron transport chain. By using multiple substrates and inhibitors of the respiratory chain complexes in intact and permeabilized cells, perturbations of inner or outer mitochondrial membrane integrity or decreased function of specific enzyme complexes as well as differences in cellular energy demand can readily be detected. No such differences could be detected following acoustophoretic processing in the present study suggesting that acoustophoresis is a gentle cell handling technology, not causing any direct effect on essential mitochondrial function or indirect effects on blood cell energy homeostasis.

Exposing cells to ultrasound standing wave conditions in microfluidic systems can be harmful to the cells [40]. This deleterious non-thermal effect of ultrasound is thought to be related to acoustic cavitation. Other non-thermal acoustic effects, such as acoustic radiation force and acoustic streaming is known to be less detrimental. Cavitation is a phenomena where collapsing gas bubbles can form jet streams under strong acoustic wave exposures (typically <1 MHz, reviewed in [14] that can punctuate/rupture the cell membranes and thereby induce cell damage. It should be noted that in this study, cells spend a very short time in the high pressure zone of the standing wave along the side walls and migrate to the central pressure minima in the middle of the microchannel where the pressure variations are zero and cavitation is unlikely to occur.

Long-term acoustic exposure times used for trapping cells have typically demonstrated no alterations in cell viability. For example, Wang et al. used hybridoma cells that were acoustically trapped up to 6 min with maximum 0.24 J/m^3^ without detecting any changes in cell viability [41]. Hultström and coauthors showed normal cell growth in COS-7 cells, a fibroblast-like cell line from monkey kidney, following 75 min of exposure to 0.85 MPa (125 J/m^3^) [42]. Applying similar acoustic energy density, they trapped human B cells for 3 days, demonstrating unaltered cell division [43]. In another study, Bazou and colleagues studied human liver carcinoma cells (HepG2) in an acoustic trap at 125 J/m^3^ for 1 min and 14 J/m^3^ for 4 min without affecting cell death and proliferation [19]. In a recent study exploring subtle cellular alterations, Bazou et al. demonstrated gene expression analysis of mouse embryonic stem cells levitated in a ultrasound standing wave trap for 60 min at acoustic pressure of 125 J/m^3^ without modifying pluripotency and gene expression [44]. Using high acoustic energy densities of 12 800 J/m^3^ for 15 min, Yasuda and colleagues focused erythrocytes at the pressure node without detecting any leakage of potassium ions indicative of cell injury [45]. Although most studies report a lack of acute adverse effect of ultrasound, Radel et al. exposed yeast cells to <150 J/m^3^ for 2 h and discovered morphological changes related to the integrity of the cell vacuole, which could be important for long-term studies [46]. These findings were, however, observed for buffer systems (ethanol/water mixture, 12%) where the yeast cells were not confined to the pressure nodal plane but rather in the interstitial space. The presence of ultrasound contrast agents can also affect the cell viability at relatively small acoustic energy densities (<150 J/m^3^) [47].

Comparing these long-term acoustic exposures with our acoustophoresis systems where we typically use actuation voltage of 10 V_pp_ (47 J/m^3^) and an acoustic exposure time of 1 second for efficient cell sorting, suggest that the present microfluidic system provide a very gentle platform for cell sorting.

In conclusion, our findings suggest that microchannel acoustophoresis can be used for effective cell handling without having any detectable effect on cell viability, inflammatory status, functional phenotype or mitochondrial respiration.
